# Music therapy with adult burn patients in the intensive care unit: short-term analysis of electrophysiological signals during music-assisted relaxation

**DOI:** 10.1038/s41598-024-73211-3

**Published:** 2024-10-09

**Authors:** Jose Cordoba-Silva, Rafael Maya, Mario Valderrama, Luis Felipe Giraldo, William Betancourt-Zapata, Andrés Salgado-Vasco, Juliana Marín-Sánchez, Viviana Gómez-Ortega, Mark Ettenberger

**Affiliations:** 1 Department of Biomedical Engineering, University of Los Andes, Bogotá, Colombia; 2 Department of Social Management, Music Therapy Service University Hospital Fundación Santa Fe de Bogotá, Bogotá, Colombia; 3 SONO – Centro de Musicoterapia, Bogotá, Colombia; 4 Burn Unit, University Hospital Fundación Santa Fe de Bogotá, Bogotá, Colombia

**Keywords:** Music therapy, Burn patients, Intensive care unit (ICU), Electroencephalogram (EEG), Electrocardiogram (ECG), Electromyogram (EMG), Medical research, Outcomes research, Rehabilitation

## Abstract

Burn patients often face elevated pain, anxiety, and depression levels. Music therapy adds to integrative care in burn patients, but research including electrophysiological measures is limited. This study reports electrophysiological signals analysis during Music-Assisted Relaxation (MAR) with burn patients in the Intensive Care Unit (ICU). This study is a sub-analysis of an ongoing trial of music therapy with burn patients in the ICU. Electroencephalogram (EEG), electrocardiogram (ECG), and electromyogram (EMG) were recorded during MAR with nine burn patients. Additionally, background pain levels (VAS) and anxiety and depression levels (HADS) were assessed. EEG oscillation power showed statistically significant changes in the delta (*p* < 0.05), theta (*p* = 0.01), beta (*p* < 0.05), and alpha (*p* = 0.05) bands during music therapy. Heart rate variability tachograms high-frequencies increased (*p* = 0.014), and low-frequencies decreased (*p* = 0.046). Facial EMG mean frequency decreased (*p* = 0.01). VAS and HADS scores decreased − 0.76 (*p* = 0.4) and − 3.375 points (*p* = 0.37) respectively. Our results indicate parasympathetic system activity, attention shifts, reduced muscle tone, and a relaxed state of mind during MAR. This hints at potential mechanisms of music therapy but needs to be confirmed in larger studies. Electrophysiological changes during music therapy highlight its clinical relevance as a complementary treatment for ICU burn patients.

**Trial registration**: Clinicaltrials.gov (NCT04571255). Registered September 24th, 2020. https//classic.clinicaltrials.gov/ct2/show/NCT04571255.

**Supplementary Information:**

The online version contains supplementary material available at 10.1038/s41598-024-73211-3.

## Background

Severe burns represent a significant global healthcare challenge, with approximately 11 million cases per year requiring medical attention worldwide^1^. Beyond physical trauma and a mortality rate of around 3%, burns have far-reaching implications for mental health and overall well-being^2,3^. A previous study found that even one year after hospital discharge, 68.5% of burn patients still experienced moderate pain, and 42.1% showed elevated anxiety and depression levels^4^. This is crucial since both continuous pain experiences and mental health impairments can pose obstacles to successful rehabilitation^5–7^.

Burn injuries lead to a dysregulated inflammatory and stress response, primarily driven by a prolonged sympathetic nervous system activation^3,8,9^. This response triggers systemic effects through the release of inflammatory mediators and stress hormones^3^. After 72–96 h post-burn injury, patients enter a hypermetabolic state that can last up to 36 months^10^. This state is characterized by elevated blood pressure, insulin resistance, muscle breakdown leading to increased protein loss, and higher resting energy expenditure^10,11^. While the precise mechanism remains unclear, it is known that stress-inducing hormones like catecholamines, glucocorticoids, glucagon, and dopamine released from the brain play a significant role in triggering this condition^3^. Overall, burn injuries can lead to a complex and prolonged hypermetabolic state driven by the dysregulated inflammatory and stress response, which can result in significant changes in the brain, muscles, and heart’s physiological activity.

In the Intensive Care Unit (ICU), music therapy is a complementary and non-pharmacological treatment provided by a trained music therapist. Music therapists address the physical, emotional, mental, social, and spiritual needs of patients to improve health, well-being, and quality of life. Music therapy has shown promising results in alleviating stress, anxiety, and depression levels, insomnia, as well as positively influencing pain perception in ICU patients^12–16^. In terms of pain control mechanisms, music therapy aligns with well-recognized theories, such as the gate control theory, which explains the processing of pain signals within the nervous system^17^. Moreover, autonomic nervous system regulation is believed to play a significant role in mediating the therapeutic effects of music therapy in a variety of medical populations^18^.

Existing evidence suggests that music therapy and other music-based interventions can effectively contribute to pain management and promote healing and recovery in burn patients. Recent meta-analyses report a statistically significant reduction in pain and anxiety levels, as well as greater relaxation^17,19^. However, it is important to note that more rigorous and high-quality randomized controlled trials (RCTs) are necessary to establish definitive conclusions regarding the efficacy of music therapy with this population^17,19,20^. Moreover, limited or no articles have examined the effects of music therapy on brain, heart, and muscle electrophysiological activity in burn patients admitted to the ICU.

This article reports a sub-analysis conducted as part of an ongoing study on the effects of music therapy with burn patients on perceived pain, mental health, vital signs, and medication usage in the ICU^21^. While the electrophysiological data obtained was an exploratory and secondary outcome measure in the study, we hypothesized that during music therapy, there would be (1) an increase in occipital Alpha and Theta brain waves, which are associated with inward-focused states of mind, (2) changes in the LF/HF ratio indicating a parasympathetic activation, and (3) a reduction in muscle tone.

## Methods

### Experimental design

This sub-analysis forms part of an ongoing single site Randomized Clinical Trial including 82 adult burn patients admitted to the ICU. Participants were 1:1 randomized to an intervention group (standard care plus a maximum of six Music-Assisted Relaxation (MAR) sessions provided by a certified music therapist over 2 weeks) or a control group (standard care only).

In this within-subject repeated measures sub-analysis, all participants were from the intervention group and received MAR, with no control participants included. Our objective was to observe patients’ responses before, during, and after MAR. Each participant experienced all conditions (pre-, during, and post-intervention) allowing observation of individual changes when intervention conditions changed. As the focus of this study was on individual responses rather than group comparisons, electrophysiological measures were not performed in the control group.

### Participants

Participants were 9 adult burn patients hospitalized in the burn unit of the University Hospital Fundación Santa Fe de Bogotá (FSFB), corresponding to 21.9% of the intervention group participants of the main trial. Participants were selected by convenience sampling, as participation in the electrophysiological measurements was voluntary and was approved on a case-by-case basis by medical staff.

### Inclusion and exclusion criteria

The inclusion criteria were burn patients of legal adult age with an expected hospitalization period longer than 7 days. Exclusion criteria were patients with known psychiatric disorders; cognitive disabilities; sedation; mechanical ventilation as well as patients with head, face, or neck burn injuries, as electrode application would not have been feasible with such injuries.

### Ethics approval and informed consent

This study was approved by the ethics committee of the University Hospital Fundación Santa Fe de Bogotá (FSFB) (CCEI-11234-2019/CCEI-11971-2020) and is registered at Clinicaltrials.gov (NCT04571255). All participants and two additional witnesses signed an informed consent. The study protocol has been published elsewhere^21^.

### Music therapy procedures

The following description of the MAR follows the CONSORT guidelines for reporting music-based interventions^22^. MAR is a music therapy technique that aims at enhancing deep relaxation of patients. MAR incorporates improvised live music, guided relaxation, and imagery based on entertainment principles^23,24^. MAR has been shown to be effective for diverse populations, including individuals with chronic pain^25,26^.

At the start of the session, participants were verbally invited to either close their eyes or focus on a fixed point, fostering their body awareness and focusing on their breathing. They were then encouraged to visualize a personal “safe place” or a favourite landscape (e.g., a forest, a mountain, a beach) while breathing with the music. Musical instruments included an acoustic guitar (Yamaha C-40), an ocean drum that replicates the sound of waves, and a Samafon. A Samafon is a non-trademarked instrument produced by Centro Vibro and consists of tuned aluminium tubes of different sizes, which hang on rope and are struck by a soft mallet, producing long-lasting, and sustained single or combined tones. The music played during the session was improvised and selected by the music therapist with the aim to induce a relaxation response in the patient and to create a serene and calm atmosphere. In general, the music had a slow and stable tempo, simple harmonic progressions (e.g., I-IV-I, I-VI-IV-V), and fluid, descendent melodies, using the middle register of the instruments, and avoiding large intervals. The volume was kept below 65 dB(A), as the ICU rooms are equipped with a ‘sound ear’ (a sound meter in the form of an ear that changes from green to yellow or red when surpassing a threshold of 65dB). of. As the MAR session concluded, the music therapist let the music slowly fade out and participants were verbally guided back to a state of increased awareness, followed by an opportunity for open dialogue to explore and reflect upon their experiences during the session. A maximum of six MAR sessions were offered throughout a maximum of two weeks and each session lasted on average 44 min, with an average of 23 min dedicated to the music itself. While treatment fidelity was not evaluated in this study, a follow-up sheet was filled out by the music therapist after each intervention including session duration, instruments used, type of imagery selected by the patient, among others.

### Electrophysiological recordings setup

Electrophysiological measurements were performed with each patient during two MAR sessions on two different days. The recordings consisted of three phases: First, in the pre-intervention (PRE) period, the resting state was measured as a baseline with the patient’s eyes closed or staring at a fixed point. Then, the music therapy intervention (MTI) was delivered by the music therapist. Finally, a post-intervention (POST) period was measured during the patient’s reincorporation after MAR. The duration of each phase varied from patient to patient due to the dynamic and limited nature of ICU time. Although we aimed for at least 5 min for PRE and POST phases and 15 min for MTI, interruptions and patient-related factors sometimes led to shorter or longer recording periods. All recordings were performed with the Micromed LTM64 (Treviso, Italy) equipment with a sampling frequency of at least 256 Hz. The Micromed LTM64 is of clinical quality and has the due approval of the Colombian National Institute for Drug and Food Safety (20090486-2015).

**Fig. 1:**
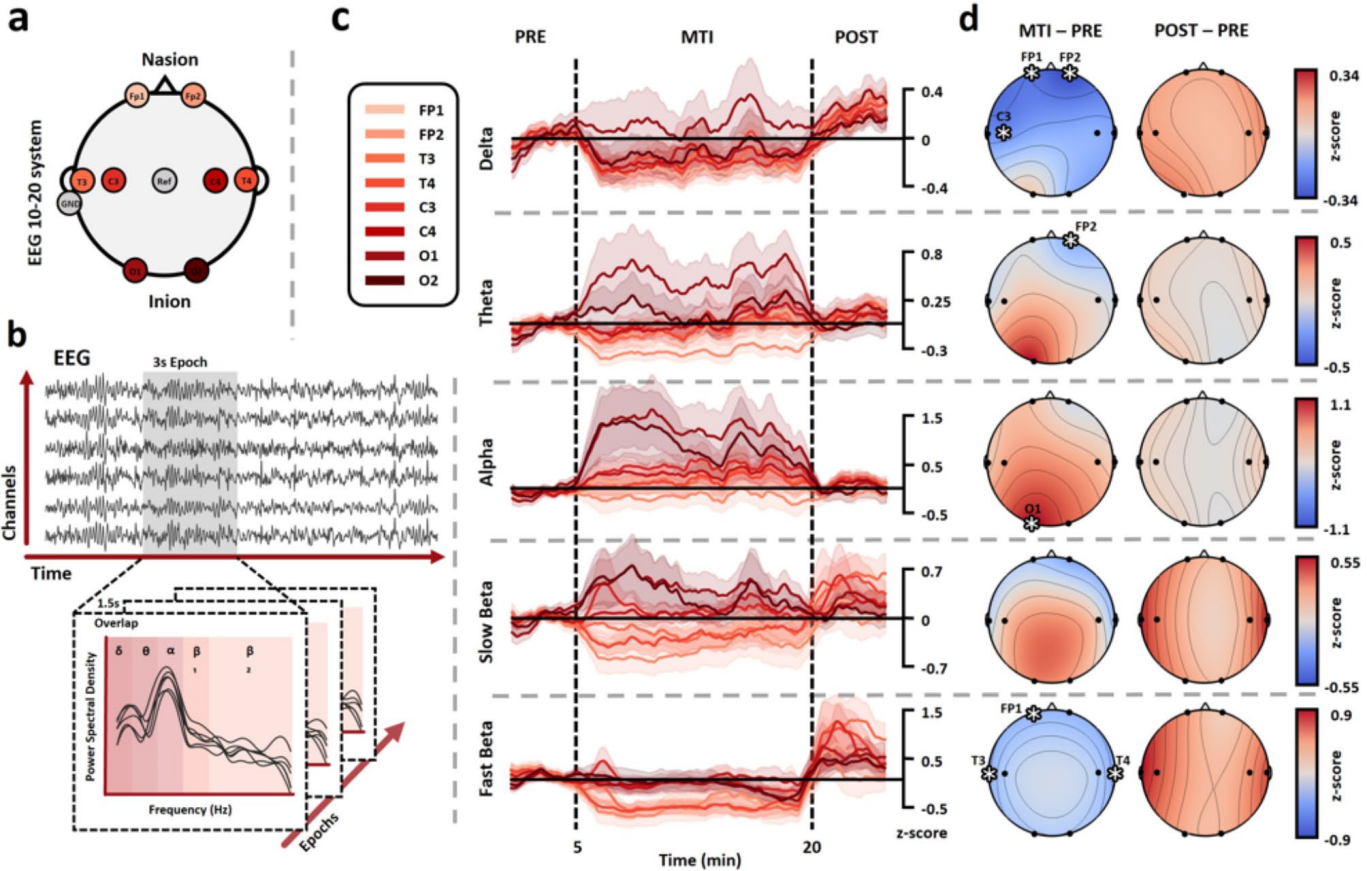
Frequency analysis for EEG. (**a**) Electroencephalogram following 10–20 international system was reduced to 8 electrodes. (**b**) One patient example of data segmentation into epochs of 3 s with an overlap of 1.5 s. For each epoch, power spectral analysis was performed, and the spectrum was divided into 5 frequency bands. (**c**) Time evolution of the power z-scores in all frequency bands is presented in rows 1 to 5, respectively (refer to the "Methods" section for frequency band range values). For each frequency band, the eight channels were plotted, with corresponding color labels displayed in the upper left corner. The colored lines represent the mean power value across all recordings for each channel in the time series. The shadow of the line corresponds to the standard error of all patients in the time series. The vertical dashed lines indicate the recording periods. (**d**) Topographic plots showing the changes in power spectral densities for all frequency bands are presented in rows 1 to 5, respectively. The left plot represents the average change across all patients between MTI and PRE, while the right plot represents the average change between POST and PRE. Consequently, values are relative to RPE, where increases are represented with red colors, and decreases are represented with blue colors. Significant changes (FDR corrected, p-values < 0.05) are marked with an asterisk (*) and labeled with the corresponding electrode name.

For the EEG setup, the electrode montage followed the international system 10–20, although to reduce the setup time for patients due to their medical condition, the number of electrodes was reduced to eight: FP1, FP2, T3, T4, C3, C4, O1, and O2. The reference electrode was set to Cz, and the ground electrode was placed on the mastoids (Fig. 1a). The ECG was acquired by a bipolar assembly of lead II with two electrodes located bilaterally in the upper part of the thorax or both arms according to the possibilities or limitations of each patient and their state of health. For the facial EMG, a bipolar electrode configuration was positioned on the left eyebrow to assess the motor activity of the corrugator supercilii muscle. The electrodes were placed with a 20 mm distance between them, following the natural alignment of the muscle fibres. This positioning ensures more precise results and minimizes potential interference from neighbouring muscles^27^.

### Background pain and anxiety-depression levels

Background pain was assessed with a Visual Analog Scale (VAS), a scale divided into 10 evenly spaced intervals of 1 cm ranging from 0 (indicating no pain) to 10 (representing the maximum pain possible). The VAS was administered after obtaining informed consent and before and after each intervention. For anxiety and depression levels, the Colombian version of the Hospital Anxiety and Depression Scale (HADS) was used^28^. The HADS consists of two sub-scales for anxiety and depression, each containing seven items. Items are rated from 0 to 3 on a four-point Likert scale. Higher scores indicate higher anxiety or depression levels, with a maximum score of 21 for each respective subscale. The HADS was administered after obtaining informed consent and subsequently after the third and last MAR session.

### Signal processing analysis

All analysis and graphics were performed using Python 3.7 with custom scripts in a combination of NumPy^29^, Pandas^30^, SciPy^31^, Matplotlib^32^, MNE^33^, Biopsy^34^, neurokit2^35^, and Visbrain^36^ libraries.

#### EEG analysis

Data was first filtered between 1 and 30 Hz by applying a zero-phase bandpass IIR filter. Then, noise and artifacts of the data were visually inspected and removed in all channels. Subsequently, an independent component analysis (ICA) algorithm from the MNE Python library removed 1 noise-detected component from the 8 channels associated with blinking and eye muscular activity. Finally, from the clean data, a central window of 5 min, 15 min, and 5 min was extracted for each patient for PRE, MTI, and POST periods, respectively.

The data was then segmented into epochs of 3 s with an overlap of 1.5 s. Power spectral density (PSD) using the Welch method was conducted on each epoch, calculating the mean power for five frequency bands: Delta (1–4 Hz), Theta (4–8 Hz), Alpha (8–12 Hz), Slow Beta (12–18 Hz), and Fast Beta (18–30 Hz) (Fig. 1b). Band power was calculated by integrating PSD values in each frequency band using Simpson’s rule. Then, the power evolutions for each frequency band and channel were z-score normalized to the PRE. Subsequently, average z-scores were calculated for each period and then compared between PRE, MTI, and POST. Results for all electrodes in each frequency band were visualized through a topographical plot of the brain using the Visbrain Python library^36^.

#### ECG analysis

**Fig. 2:**
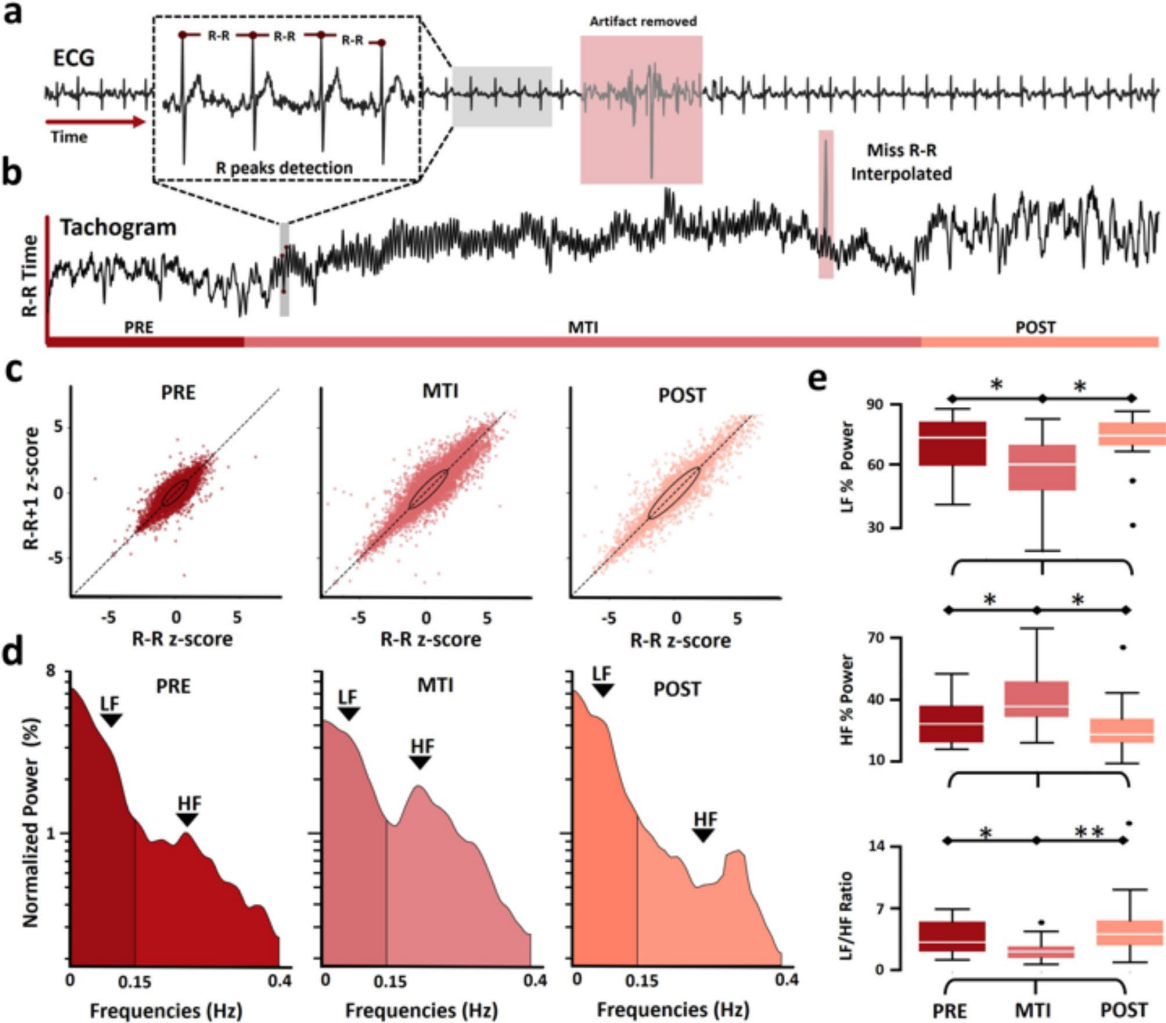
Time, Non-linear, and frequency analysis for ECG. (**a**) ECG example of artifact remotion and R peak detection. (**b**) Tachogram extraction example for patient 3 session 1. R-R time segments were computed in the time series, miss detections were excluded, and then interpolated. (**c**) Normalized Poincaré plot for PRE, MTI, and POST for all patients. Confidence ellipses were plotted with SD1 as perpendicular radius and SD2 as parallel radius to the identity lines, represented by dashed lines. (**d**) Power spectral density plots average across all patients for PRE, MTI, and POST. The three plots are logarithmic scale and share the same axes. (**e**) Boxplots to compare PRE, MTI, and POST. The plots are arranged from top to bottom as follows: LF, HF, and the LF/HF ratio. Black lines at the top indicate significant differences between periods (*p-value < 0.05, **p-value < 0.01).

Data was first filtered between 1 and 40 Hz. Then, the noise and artifacts of the data were automatically removed and manually corrected (Fig. 2a). The R-R intervals were calculated, and intervals exceeding 1.5 s or falling below 0.3 s were excluded. The remaining intervals were interpolated to correct for potential errors. To the clean data, a central window of 5 min, 15 min, and 5 min was extracted for PRE, MTI, and POST periods, respectively (Fig. 2b).

Heart rate variability (HRV) measures from the successive R-R values were calculated. Mean of R-R Interval (meanRR), standard deviation of R-R Intervals (STDRR), and percentage of R-R Intervals differing by more than 50 ms (pRR50) were computed for the PRE, MTI, and POST periods.

Autonomic activity was visualized using a non-linear representation of the Poincaré plot^37^. Tachograms were normalized by z-scores based on each patient’s PRE values. The z-score R-R values were plotted against the subsequent z-score RR + 1 values, resulting in a normalized Poincaré plot. SD1 and SD2 were calculated from the Poincaré plot, with SD1 representing the short-term variability perpendicular to the line of identity, while SD2 represents long-term variability along the line of identity^38^.

To conduct frequency analysis of the tachogram, cubic splines interpolation was applied to re-sampling at 4 Hz frequency. PSD analysis using the Welch method was performed on the complete interpolated central window extracted for each period. This analysis requires a minimum recommended time window of 5 min to examine the lowest frequency of interest, 0.04 Hz, accurately^39^. The PSD was normalized by dividing each value by the total power. The average power for low-frequency (LF) (0.04–0.15 Hz) and high-frequency (HF) (0.15–0.4 Hz) was calculated using Simpson’s rule. Finally, the average power for PRE, MTI, and POST were compared for each frequency band and the ratio LF/HF.

#### EMG analysis

Data was initially filtered between 20 Hz and 200 Hz. Frequencies below 20 Hz were eliminated to minimize motion artifacts and blinking, following recommended guidelines for facial muscle analysis^40^. Frequencies above 200 Hz were excluded. Therefore, only recordings with a sampling rate of at least 512 Hz were analysed for EMG to comply with Nyquist’s theorem. This ensures that the sampling rate is at least twice the frequency being analysed, ensuring accurate frequency analysis. A notch filter was subsequently applied to remove 60 Hz electrical noise and its harmonics. Any remaining noise and artifacts in the data were carefully inspected and removed from all channels. Finally, a central time window of 5 min for the PRE, 15 min for MTI, and 5 min for POST periods were extracted for each patient.

**Fig. 3:**
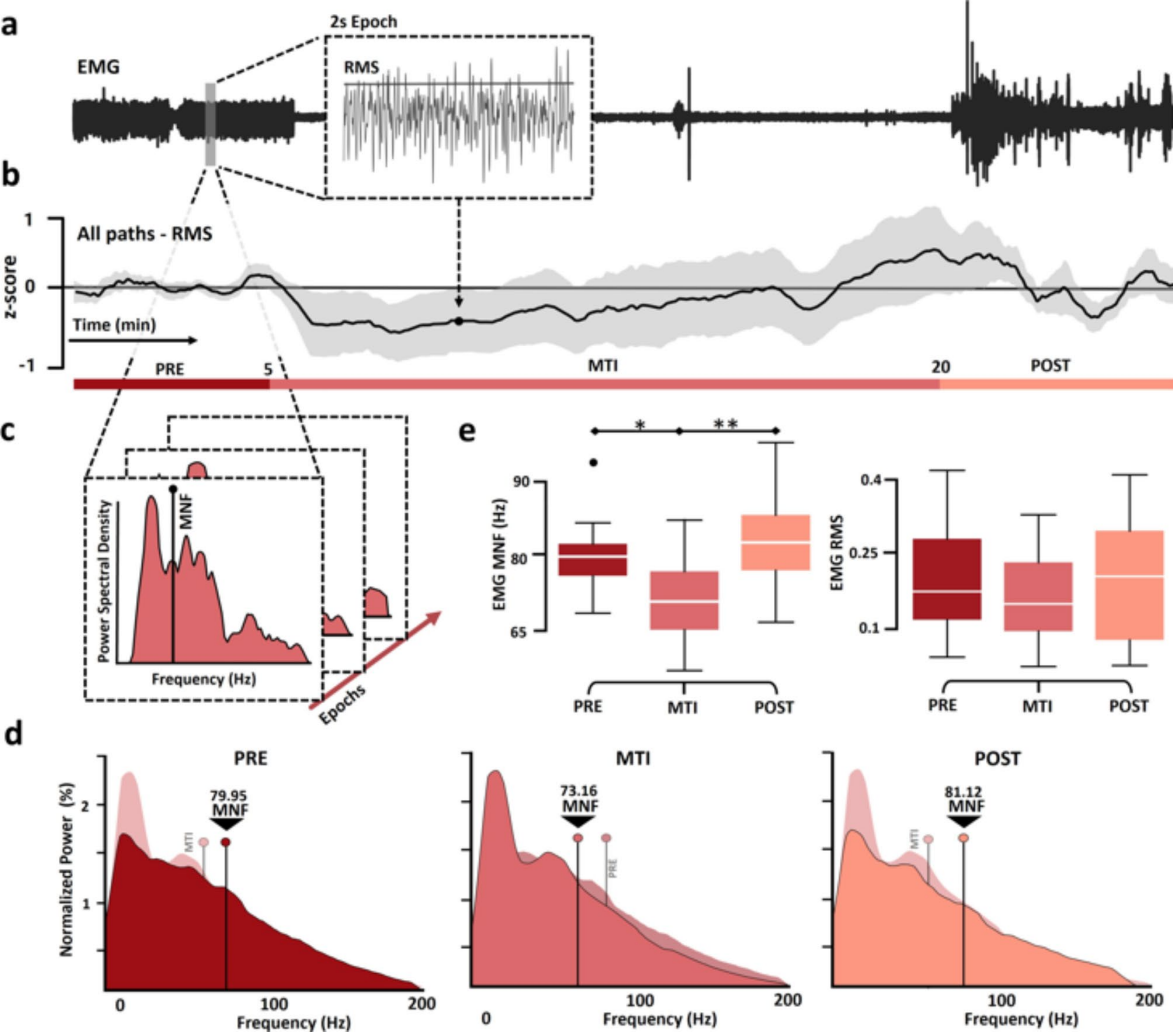
Amplitude and frequency analysis for EMG. (**a**) EMG example for patient 3 sessions data segmentation into epochs of 2 s with an overlap of 1 s (**b**) Time evolution of all patients average RMS calculated for each epoch. The shadow of the line corresponds to the standard error of all patients in the time series. (**c**) For each epoch, power spectral analysis was performed, and the mean frequency was calculated. (**d**) EMG average normalized power spectral density across all patients for PRE, MTI, and POST. The average mean frequency across patients is marked with a black line. The behind shadow plot represents another period power spectral analysis. The three plots share the same axes. (**e**) Box plots summarizing the distribution of MNF and RMS values obtained from individual recordings during the MT intervention. Black lines indicate significant differences between periods (*p-value < 0.05, **p-value < 0.01).

The cleaned data was then segmented into epochs of 2 s, with a 1-second overlap. To measure changes in amplitude, the Root Mean Square (RMS) was computed for each epoch. The time series RMS values were normalized using z-scores in relation to PRE, enabling comparisons between different recordings^41^ (Fig. 3a).

Then, to analyse frequency components, PSD using the Welch method was conducted on each EMG epoch. Mean PSDs were calculated for the PRE, MTI, and POST periods. To normalize the PSDs of each period, each spectrum was divided by total power. Finally, as a measure of muscle motor unit recruitment and muscle tension, the mean frequency (MNF) was calculated for each PSD period^42,43^ (Fig. 3c).

### Statistical analysis

Significant differences between PRE, MTI, and POST periods were independently analysed for each comparison across all patients and recordings. Considering the nature of the data, the nonparametric permutation test was chosen for analysis, considering the flexibility of the test statistic with minimal assumptions required for validity. Permutation test was performed to compare two populations, with a total of 1600 permutations^44^. The test probed the equality of means between PRE, MTI, and POST periods for each feature calculated for EEG, ECG, and EMG across all patients and recordings. For the VAS scores, the PRE and POST periods were compared, while for the HADS scores, the baseline and last measured values were compared across all patients. The null hypothesis: $\:{H}_{0}:\:{\mu\:}_{1}-{\mu\:}_{2}=0,$ was rejected when the p-value was less than 0.05. Multiple comparisons were controlled by the Benjamini-Hochberg false discovery rate (FDR)^45^ across all electrodes in EEG measures for each frequency band. This was performed to correct for possible type 1 errors in the results. All analysis was performed using Python 3.7 custom scripts.

## Results

### Recording and participants

A total of 17 electrophysiological recordings were taken from 9 ICU burn patients during MAR, with two separate recordings performed on two different days for each patient. One patient had only one recording because he was unexpectedly discharged from the hospital after the first session. The age range of patients was 18 to 65, with a mean age of 33.6 years. 8 out of the 9 patients were males (88.9%). The two main causes of burns among the nine participants were thermal or electrical, with severity ranging from first to second degree. All patients met the inclusion and exclusion criteria. The socio-demographic and medical data for each patient can be found in Table 1.

**Table 1:** Socio-demographic and medical data.

ID	Age (y)	Sex	Burn Type	Severity
1	36	Male	Thermal	1
2	20	Male	Thermal	1
3	65	Male	Electric	2
4	29	Male	Thermal	1
5	18	Female	Thermal	1
6	19	Male	Thermal	1
7	58	Male	Electric	2
8	28	Male	Thermal	1
9	29	Male	Electric	2

*ID* Patient indicator. Severity: Severity of the burn on a 3-point scale going from first to third degree. Medication: Pain medication of the patient during the day of the music therapy session.

On average, PRE lasted for 9.12 min (range 4.73 to 15.0 min), MTI lasted for 21.49 min (range 8.03 to 29.81 min), and POST lasted for 8.71 min (range 3.36 to 20.71 min). Periods shorter than the expected time were included in the analysis in their entirety. For ECG frequency analysis specifically, if a window was less than 5 min, we increased the lower frequency cutoff (0.04 Hz) proportionally to ensure a minimum 12 oscillation cycle, as recommended^39^.

### EEG

In the delta band, a statistically significant decrease in power was noted across the prefrontal and left central leads. The mean change was for Fp1=-0.27 (SD = 0.37, FDRp = 0.02), for Fp2=-0.34 (SD = 0.31, FDRp = 0.005), and for C3=-0.23 (SD = 0.35, FDRp = 0.036). In the theta band, a significant decrease in power was observed in the right prefrontal lead, Fp2, with a mean change of -0.31 (SD = 0.3, FDRp = 0.01). For the alpha band in the O1 lead, there was an increase of 1.1 (SD = 1.8, FDRp = 0.05). Lastly, in the fast beta band, decreases were noted in the left prefrontal lead, Fp1, with a change of -0.5 (SD = 0.35, FDRp = 0.002), and in both temporal leads, T3 and T4, with changes of -0.58 (SD = 0.49, FDRp = 0.002) and − 0.57 (SD = 0.52, FDRp = 0.002), respectively. No significant changes were observed in any frequency band during the POST compared to the PRE (Fig. 1d).

### ECG

ECG recordings were taken in 16 out of 17 MAR sessions. One patient’s data was excluded due to a lead disconnection incident. During the POST, a significant increase in STDRR was observed, with an increase of 17.64 ms (SD = 28.27 ms, *p* = 0.017) compared to PRE (Table 2). Comparable results were observed for SD2 in the Poincaré plots (Fig. 2c). In the POST, there was a significant increase compared to the PRE, with a z-score change of 0.576 (SD = 0.831, *p* = 0.011) and a z-score increase of 0.52 (SD = 0.6 ms, *p* = 0.031) compared to the MTI.

**Table 2:** Frequency, HRV, and Nonlinear ECG Parameters – Comparison among the three periods: PRE, MTI, and POST.

	MeanRR (ms)	STDRR (ms)	pRR50 (%)	SD1	SD2	SD1/SD2	LF (%)	HF (%)	LF/HF
MTI-PRE									
Average change	15.75	0.307	0.017	-0.009	0.05	-0.019	-13.93	13.93	-2.02
SD. Change	30.66	16.23	6.106	0.131	0.45	0.077	15.61	15.61	3.14
p-value	0.74	0.95	0.997	0.91	0.613	0.68	0.026*	0.021*	0.013*

	MeanRR	STDRR	pRR50	SD1	SD2	SD1/SD2	LF	HF	LF/HF
POST-PRE									
Average change	-3.38	17.64	0.626	0.083	0.576	-0.04	2.71	-2.71	0.98
SD. Change	49	28.27	8.00	0.21	0.831	0.119	18.82	18.82	6.28
p-value	0.927	0.017*	0.903	0.39	0.011*	0.36	0.622	0.668	0.57
MTI-POST									
Average change	19.13	-17.34	-0.609	-0.07	-0.52	0.024	-16.64	16.64	-3.01
SD. Change	47.2	20.26	7.92	0.153	0.6	0.086	15.58	15.58	4.77
p-value	0.68	0.132	0.915	0.478	0.031*	0.58	0.011*	0.013*	0.0006*

The data presented represent the average change and the standard deviation of the changes for the 17 recordings. The value represents the difference between the first period and the second period in the title. For instance, in MTI-PRE, a positive value indicates an increase in the variable during MTI compared to PRE. Significance was determined using a permutation test, where p-values < 0.05 indicated significant changes and were marked with an *

Regarding the frequency analysis of the tachogram, the findings revealed statistically significant average increases in HF and decrease in LF, as well as the LF/HF ratio, across all recorded sessions during MTI when compared to both the PRE and POST (all *p* < 0.05). No statistical differences were observed in HF, LF, or the LF/HF ratio between the PRE and POST periods. Other parameters, including meanRR, pRR50, SD1, and SD1/SD2, did not exhibit statistical significance (Table 2) (Fig. 2d and e).

### EMG

Of the 17 sessions analysed, 14 recordings were included for analysis. Two recordings were excluded due to technical difficulties with the EMG signal acquisition, and one was excluded because its sample rate was under 512 Hz. Statistically significant reductions were observed in the value of MNF during the MTI across all recordings, as compared to PRE and POST. Specifically, during MTI, MNF showed a decrease of 6.49 Hz (SD = 6.2 Hz, *p* = 0.01) compared to PRE, followed by an increase of 7.96 Hz (SD = 7.3 Hz, *p* = 0.003) during POST. The MNF values between PRE and POST did not exhibit any significant differences. No statistical differences were observed in the RMS amplitude analysis ratio between PRE, MTI, and POST periods (Fig. 3b and e).

### Pain levels

For this study, only the VAS scores of the electrophysiological recording sessions have been analyzed. The average VAS score across all nine patients was 2.0 for PRE (Range: 0 to 8, SD = 2.47) and 1.24 for POST (Range: 0 to 7, SD = 2.18). On average, there was a decrease of 0.76 in the pain level. (Range: 4 to -2, SD = 1.55). No statistically significant differences between PRE and POST measurements were found (*p* = 0.42) (Table 3).

**Table 3:** Pain, anxiety, and depression levels.

ID				VAS						HADS
	First recording		Second recording		Anxiety		Depression		Total	
	PRE	POST	PRE	POST	First	Last	First	Last	First	Last
1	0	0	7	4	9	4	7	2	16	6
2	3	0	4	6	9	8	3	2	12	10
3	0	0	0	0	2	2	3	0	5	2
4	2	0	8	7	7	6	7	1	14	7
5	3	2	0	1	11	9	6	9	17	18
6	0	0	0	0	7	1	4	1	11	2
7	2	0	0	0	0	1	0	2	0	3
8	1	0	4	0	2	1	0	0	2	1
9	0	1	–	–	4	–	2	–	6	–

Average across patients										
			VAS		A-HADS		D-HADS		T-HADS	
			PRE	POST	First	Last	First	Last	First	Last
Total			2.00	1.24	5.875	4.00	3.75	2.125	9.625	6.125

Changes across patients										
				VAS	A-HADS		D-HADS		T-HADS	
Average change				-0.764		-1.875		-1.625		-3.5
SD. Change				1.55		2.29		2.99		4.41
p-value				0.42		0.359		0.312		0.275

Individual results per patient for VAS and HADS. Vas was taking pre-music therapy intervention (PRE) and post-music therapy intervention (POST). HADS was assessed before the first music therapy intervention and then after the third and sixth music therapy intervention**.** HADS presented was based on the latest measure acquired for the total music therapy intervention of each patient.

*VAS* Visual Analogue Scale for Pain, *HADS* Colombian version of the Hospital Anxiety and Depression Scale, *A* anxiety, *D* depression, *T* Total. *ID* Patient indicator.

### Anxiety and depression levels

HADS values were measured for eight patients, excluding one patient who left the hospital before the last HADS timepoint and thus had only baseline measures. The depression HADS subscale was 3.750 before the first music therapy session and 2.125 at the last session. The average change was − 1.625 (SD = 2.99, *p* = 0.312). For the anxiety HADS subscale, the first music therapy session average value was 5.875 before and at the last session. The average change was − 1.875 (SD = 2.29, *p* = 0.359). The total mean HADS score was 9.625 for baseline (Range: 17 to 0, SD = 6.06) and 6.125 for the last session (Range: 18 to 0, SD = 5.89), with a decrease of 3.5 points (Last – First: Range: -7 to 1, SD: 3.33, *p* = 0.275). No statistically significant differences were found in the subscales or the total HADS scales (Table 3).

## Discussion

This study aimed to investigate the effects of MAR in electrophysiological recordings in 9 burn patients hospitalized in the ICU. The results show that during the intervention, patients experienced a statistically significant decrease in delta and theta bands in the prefrontal areas, while the beta band was reduced in the frontotemporal areas. Alpha band activity increased in the occipital area (Fig. 1c and d). However, it is worth noting that some baseline recordings were done with eyes open, which raises the possibility that the increase in alpha in the occipital region could be due to the act of closing the eyes^46^. This is because during MAR, patients are given the option to close their eyes or leave them open. The reason for this is that burn injuries are often a very traumatic event for patients and increased sensibility during MAR might increase discomfort in patients when keeping their eyes closed. Providing patients with the option to close or leave their eyes open increases a sense of control and safety, which is important for achieving a relaxation response. Therefore, in interpreting these results, we consider not only the specific change in alpha but also other observed physiological variables and the coalescence with other frequencies^47^. The results of ECG tachograms showed that there were statistically significant average increases in the HF band and decreases in the LF band, as well as the LF/HF ratio during MTI (Fig. 2d and e). Additionally, facial EMG showed a decreased MNF during MAR (Fig. 3d and e). Although there were no significant changes in the VAS and HADS scores, patients showed a trend toward lower anxiety, depression, and background pain.

### EEG

During the MTI, common trends in EEG oscillation power were observed across patients. Changes in the PSD for different EEG frequency bands have been relevant for identifying and understanding brain biomarkers^48–50^. Music, at the same time, has been shown to modulate brain activity and has been used in therapy with people with depression, stress, and anxiety^51^. The Delta band is related to attention and motivational processes^52^. Notably, a power increase in frontal area is associated with mental tasks and internal concentration^53^. Moreover, several studies have reported increased delta activity in the frontal areas during painful stimuli^49^. Also, the processing of emotional stimuli and pain perception has been linked to theta bands in frontal areas in prior research^50,54,55^. This suggests that decreased frontal activity for delta and theta may reflect a shift in attention towards sensory inputs generated by the MAR, resulting in reduced pain perception. This could potentially hint at a sensorial-attentional mechanism of MAR generating a change of attention to the musical and sensorial stimuli. This is in line with previous studies, demonstrating a reduction in evoked pain and associated brain potentials through the implementation of distraction techniques^56^. The prefrontal area and its respective brain wave activities exhibit functional and anatomical connections with multiple brain regions, including the insula, which has a relevant role in pain processing^57^. The change in attention based on the gate control theory can reveal how changing focus from pain stimuli to music stimuli can reduce pain perception. Similar findings have been reported in the context of music therapy for pain and mental health. In a study using magnetoencephalography in healthy individuals, researchers identified a relationship between a decrease in delta power in the midcingulate cortex and anterior insula and a significant reduction in pain levels using participant preferred music^58^. Moreover, adults with experience of abandonment show decreased frontal theta during music therapy, which may improve negative feelings^59^.

Alpha and beta bands have been related to more complex cognitive processes. Particularly, it is hypothesized that the beta band plays a crucial role in sensorimotor control and predictive abilities, while alpha could actively inhibit irrelevant processes^46,47,60^. Excessive fast beta waves in adults have been found to be associated with stress and anxiety^61^. Additionally, various studies have observed that enduring or chronic pain can lead to a decrease in the Alpha frequency band, which may be linked to the severity of the pain^62^. As per our observations in the Beta and Alpha bands, there appears to be a potential alteration in the cognitive process toward a more relaxed state of consciousness. This shift may be mediated by the emotional and musical aspects, but also by the imagery used during the MAR. We suggest that MAR could encompass an emotional and physiological relaxation response elicited by musical aspects and imagery. However, we did not evaluate direct dynamic changes through brain activity; another study found that EEG activity during music therapy showed a positive emotional impact on advanced cancer patients^63^. This was correlated with reduced tiredness, anxiety, and breathing difficulties, as well as increased well-being reported by patients^63^. Our results indicate that the increased alpha related to imagery and cognitive process initiation is accompanied by the reduction in beta associated with a decrease in cognitive process, which could be interpreted as a relaxed state of mind. A similar study found that adding improvisational music therapy to standard care for people with mental health challenges significantly reduced depression and anxiety symptoms and increased alpha activity in the left frontotemporal area of the brain^64^. Another study found similar results, in which the analysis of music perception and imagery showed an increase in posterior alpha for healthy patients^65^. In general, these findings are interpreted as robust, where alpha has been shown as the most dominant effect in multiple meditation-relaxation techniques in different populations, showing an increase during relaxation and imagery processes^61,66^.

### ECG

The autonomic nervous system is regulated by signals within the central nervous system and feedback mechanisms that respond to internal and external signals^67^. The parasympathetic branch promotes relaxation and restorative processes, often counterbalancing the effects of the sympathetic system. The HF component in the tachogram frequency analysis is associated with parasympathetic activity, while the LF component is related to sympathetic activity^68^. The LF/HF ratio is often used as an indicator of autonomic balance and can provide valuable information about the balance between the parasympathetic and sympathetic systems^39^. It is essential to consider simultaneously the changes observed in HF, LF, and the LF/HF ratio^69^. In this context, the results of this study potentially indicate that MAR activated the parasympathetic branch in the patients. These results are in line with previous findings in which a lower LF/HF ratio was associated with deep relaxation during meditation^68^. Additionally, similar increases in parasympathetic activity during music therapy have been reported in previous studies for various medical conditions^70–72^. Specifically, MAR with a slow pulse has been observed to promote parasympathetic activity^70^. Moreover, the emotional influence of music is well-documented^73^, and emotions are linked to the ANS promoting relaxation^74^. Again, it is possible that the cognitive-relaxation mechanism could balance the effect of prolonged sympathetic activation in burn patients. As mentioned previously, a prolonged activation of the sympathetic nervous system is triggered by burn injuries^3,8,9^. Our results indicate that MAR promotes parasympathetic activation, which again could potentially improve mental health in this population.

### EMG

Chronic pain was found to increase muscle tone^75^, which can lead to tension-type headaches and related issues^76^. Moreover, higher muscle resting energy expenditure is a characteristic of burn inflammatory response^10,11^. MNF, as a measure associated with muscle motor unit recruitment, can provide information on non-fatigue muscle tone^43^. Therefore, the decrease observed in MNF during MTI could be related to a reduction in muscle tone during MTI. These reductions are consistent with the autonomic response patterns and the experience of pleasant auditory stimuli^77^. This could support a relaxed state of mind and an increase of the parasympathetic system relevant to counterbalancing the higher muscle resting energy driven by the prolonged sympathetic activation. On the other hand, a decreased muscle tone could have a possible effect on background pain perception^75^. This supports the sensorial-attentional mechanism driving the patient from the pain stimuli to the music. The findings of this study align with previous research reporting a decrease in muscle tension in burn patients during MAR^78^. Another study involving preferred relaxing music also found a decrease in muscle tension^79^. These findings collectively suggest that music therapy has the potential to contribute to the relaxation of muscle tension in burn patients.

### Pain, depression, and anxiety levels

Our study did not find any significant differences in background pain, depression, or anxiety levels across patients. We did observe that background pain levels in burn patients vary from one session to another. Additionally, we noticed that many participants had low initial scores. Including our small sample size, these factors limit our ability to conclude and may impact the accuracy of our statistical results. Additionally, the setup time for the electrophysiological measures meant an additional effort for patients and might have thus impacted pain perception. Nevertheless, common trends were observed that could potentially indicate improvement in mental health. Minimal Clinically Important Difference (MCID) is a recognized threshold that determines the smallest clinically meaningful change in subjective measures^80^. For the VAS scale, the MCID was reported to be 1.4^81^ and 1.7 for HADS^82–84^. Although there was a decrease in VAS, indicating less pain perception after the MTI, it did not reach the MCID. On the other hand, the HADS score was found to be above the threshold, potentially indicating an improvement. This is consistent with previous research that has found music therapy to be effective in reducing stress, anxiety, and depression levels, as well as improving sleep quality and pain perception in ICU patients^12–16^. However, due to the small sample size we were unable to obtain statistically significant results for the VAS and HADS metrics; however, our results align with existing evidence suggesting that music therapy can be a valuable strategy that promotes healing and recovery in burn patients, with the potential to reduce pain, stress, and anxiety levels^17,19^.

### Limitations

It is important to note that this study has several limitations. First, due to the small number of participants, the findings should be interpreted with caution and cannot be generalized. Second, there was notable homogeneity among the participants. The sample included only one female out of nine patients, with most participants aged between 18 and 36 years, and two individuals who were older, at 58 and 65 years. Thus, a conservative stance should be taken when extrapolating these results to other socio-demographic patient groups. Third, while data on medication usage will be reported in the main study, ICU patients might be exposed to multiple drugs or treatments that could have affected cardiac, muscle, or brain functioning. Fourth, the ICU environment presents various challenges and limitations for electrophysiological measurements, such as noise, interruptions, and medical equipment. These factors can cause interference and artifacts in the recorded signals. One of the principal sources of error was the time required for setup. To address this, we developed a strategy of initializing all equipment and conducting routine checks and system testing before entering the ICU room. This approach reduced setup time and allowed us to avoid rushing during electrode placement, thereby minimizing the risk of lead disconnection, artifacts, and noise. EEG cap montages are generally simpler and faster to use than free lead electrodes. However, we experience difficulties in placing the cap correctly and ensuring proper lead connections. We decided to use a free lead EEG montage consisting of only eight electrodes, as longer montage times can cause stress and anxiety in patients during the procedure. Some studies suggest that using fewer electrodes do not affect the results^85,86^, but further research is needed to determine the optimal placement and quantity for this specific environment. Moreover, we took great care to keep the lead cables organized and out of the patient’s reach to prevent any interference or discomfort. Specifically, for the frontal electrodes and EMG leads, we ensured that no cables touched the patient’s face, routing them from the forehead behind the head. This approach was intended to avoid discomfort and reduce the risk of the patient adjusting the cables, which could lead to disconnections and artifacts. Fifth, it is worth noting that some baseline recordings were done with eyes open, which raises the possibility that the increase in alpha in the occipital region could be due to the act of closing the eyes^46^. Therefore, in interpreting these results, we consider not only the specific change in alpha but also other observed physiological variables and the coalescence with other frequencies^47^. Sixth, since the focus of this study was on short-term analysis, we might have missed intra-session dynamics and long-term effects. Future research should try to increase POST-intervention time, control the quantity and frequency of the sessions, and quantify potential habituation effects. Seventh, while the electrophysiological data indicate music therapy’s potential for pain relief, the difference of the pre-post VAS scores did not reach statistical significance. While this could be due to the small sample size and/or the additional efforts patients made for participating in this study, it is nevertheless worth considering a mixed-methods research design for future studies to also understand the meaning of individual pain relief and the lived experiences of patients participating in music therapy. Finally, we did not reach a direct correlation between electrophysiological measures and the VAS and HADS. Studies designed to identify potential biomarkers correlated with improved mental health and pain levels during music therapy, both in the short and long term, are necessary. This could include inflammatory biomarkers such as catecholamines, glucocorticoids, and glucagon to have a better understanding and follow-up physiological changes during music therapy.

## Conclusions

To our knowledge, this is the first study to investigate brain oscillation during music therapy for ICU burn patients. The findings provide first insight into MAR’s potential to physiological changes that promote parasympathetic activity and guide the patient to a more relaxed state of mind during music therapy. These results support the implementation of music therapy as a complementary treatment for improving pain management and mental health in burn patients. We propose that the beneficial effects of music therapy can be attributed to the interaction of two distinct mechanisms: the sensorial-attentional mechanism mediated by the external stimulus of music and the cognitive-relaxation mechanism inducing changes in the physio-psychological states. The sensorial-attentional mechanism involves the shift in attention towards the music, diverting focus from pain stimuli and reducing pain perception. The cognitive-relaxation mechanism encompasses the emotional and physiological relaxation response elicited by the music and imagery of MAR, leading to increased parasympathetic activity and overall relaxation. Larger studies with adequate sample size and power are needed to further investigate music therapy’s potential to help burn patients on their path toward recovery and rehabilitation.

## Electronic supplementary material

Below is the link to the electronic supplementary material.

Supplementary Material 1

## Data Availability

The electrophysiological dataset is available at the data archive OpenNeuro^87^. The OpenNeuro Dataset ds004840 can be reached via https://openneuro.org/datasets/ds004840^88^. The dataset was formatted following the 1.8.0 version of the Brain Imaging Data Structure (BIDS)^89^ for EEG^90^. The Python codes made for the analysis and elaboration of graphs are available in the GitHub public repository under the MIT license: https://github.com/jgcordoba/BurnICU_MusicTherapy_Signals.

## Abbreviations

ECG: Electrocardiogram
EEG: Electroencephalogram
EMG: Electromyography
HRV: Heart rate variability
HF: High frequency
HADS: Hospital Anxiety and Depression Scale
ICU: Intensive care unit
LF: Low frequency
MNF: Mean Frequency
MCID: Minimal Clinically Important Difference
MAR: Music-Assisted Relaxation
MTI: Music Therapy intervention
PRE: Pre-intervention
POST: Post-intervention
PSD: Power spectral density
RMS: Root mean square
VAS: Visual Analogue Scale
