# Supplementary material for: Music therapy with adult burn patients in the intensive care unit: short-term analysis of electrophysiological signals during music-assisted relaxation
Source: Sci Rep. 2024 Oct 9;14:23592. doi: 10.1038/s41598-024-73211-3 (PMC11464633; doi:10.1038/s41598-024-73211-3)
Supplement: Supplementary file 1 — Supplementary Material 1 [file 41598_2024_73211_MOESM1_ESM.pdf]

Supplementary Data

**Supplementary Table I: EEG AVERAGE CHANGES IN BRAIN OSCILLATIONS' POWER BETWEEN INTERVENTION PERIODS: Mean ± Standard deviation**									
**MTI - PRE**									
**Frequency band**		**Electrodes**							
		**Fp1**	**Fp2**	**T3**	**T4**	**C3**	**C4**	**O1**	**O2**
**Delta**									
	Value	**-0.28±0.37**	**-0.34±0.31**	-0.23±0.5	-0.18±0.45	**-0.23±0.35**	-0.16±0.35	0.08±0.77	-0.14±0.55
	p-value	**0.015***	**0.005***	0.162	0.193	**0.043***	0.162	0.687	0.376
**Theta**									
	Value	-0.05±0.58	**-0.3±0.3**	-0.08±0.51	-0.05±0.42	0.08±0.77	0.02±0.37	0.59±1.24	0.18±0.82
	p-value	0.807	**0.015***	0.807	0.807	0.807	0.807	0.165	0.807
**Alpha**									
	Value	0.26±0.85	-0.09±0.77	0.19±0.92	0.18±0.72	0.45±1.04	0.34±0.8	**1.11±1.26**	0.86±1.83
	p-value	0.389	0.699	0.561	0.513	0.219	0.219	**0.050**	0.173
**Slow Beta**									
	Value	-0.27±0.62	-0.43±0.9	-0.18±0.52	-0.27±0.56	0.14±0.53	0.11±0.41	0.4±1.01	0.3±1.09
	p-value	0.242	0.242	0.312	0.242	0.363	0.363	0.261	0.363
**Fast Beta**									
	Value	**-0.5±0.69**	-0.51±1.21	**-0.58±0.49**	**-0.57±0.52**	-0.09±0.65	-0.08±0.45	-0.18±0.46	-0.16±0.57
	p-value	**0.002***	0.196	**0.002***	**0.002***	0.665	0.582	0.196	0.413
**POST - PRE**									
**Frequency band**		**Electrodes**							
		**Fp1**	**Fp2**	**T3**	**T4**	**C3**	**C4**	**O1**	**O2**
**Delta**									
	Value	0.13±0.31	0.14±0.45	0.19±0.32	0.23±0.39	0.15±0.29	0.13±0.35	0.25±0.48	0.1±0.27
	p-value	0.125	0.309	0.088	0.088	0.113	0.254	0.088	0.198
**Theta**									
	Value	0.11±0.25	0.06±0.41	0.16±0.34	0.16±0.41	0.11±0.26	0.07±0.23	0.18±0.56	-0.02±0.54
	p-value	0.264	0.680	0.264	0.264	0.264	0.390	0.390	0.932
**Alpha**									
	Value	0.11±0.32	-0.03±0.57	0.18±0.43	0.21±0.29	0.12±0.41	0.12±0.33	0.13±0.52	-0.02±0.64
	p-value	0.458	0.917	0.458	0.060	0.458	0.458	0.459	0.917
**Slow Beta**									
	Value	0.2±0.67	0.08±0.95	0.55±0.99	0.54±1.05	0.36±0.74	0.22±0.46	0.21±0.59	0.08±0.58
	p-value	0.332	0.777	0.150	0.150	0.157	0.171	0.298	0.676
**Fast Beta**									
	Value	0.43±1.17	0.17±1.45	0.9±1.6	0.73±1.33	0.72±1.67	0.49±0.92	0.33±0.8	0.3±0.59
	p-value	0.191	0.703	0.075	0.075	0.142	0.075	0.151	0.116
**MTI - POST**									
**Frequency band**		**Electrodes**							
		**Fp1**	**Fp2**	**T3**	**T4**	**C3**	**C4**	**O1**	**O2**
Delta									
	Value	**-0.41±0.5**	**-0.48±0.46**	**-0.42±0.43**	**-0.41±0.44**	**-0.39±0.39**	**-0.29±0.42**	-0.17±0.65	-0.25±0.55
	p-value	**0.008***	**0.010***	**0.020***	**0.020***	**0.008***	**0.041***	0.478	0.153
Theta									
	Value	-0.16±0.44	-0.37±0.38	-0.24±0.35	-0.21±0.45	-0.03±0.66	-0.05±0.35	0.41±0.93	0.2±0.55
	p-value	0.598	0.100	0.473	0.473	0.916	0.801	0.561	0.598
Alpha									
	Value	0.15±0.82	-0.06±0.76	0.01±0.93	-0.04±0.81	0.33±1.06	0.23±0.83	0.98±1.71	0.88±1.39
	p-value	0.869	0.971	0.971	0.971	0.679	0.679	0.250	0.250
Slow Beta									
	Value	-0.47±0.42	-0.51±0.41	-0.73±0.81	-0.81±0.92	-0.22±0.67	-0.1±0.49	0.19±0.89	0.22±0.9
	p-value	0.132	0.258	0.055	0.055	0.535	0.535	0.535	0.535
Fast Beta									
	Value	**-0.93±1.03**	-0.68±1.36	**-1.47±1.51**	**-1.3±1.38**	-0.81±1.34	**-0.57±1.02**	**-0.52±0.85**	-0.46±0.7
	p-value	**0.025***	0.173	**0.002***	**0.002***	0.102	**0.048***	**0.048***	0.055

Supplementary Table I: EEG average changes in brain oscillations' power between intervention periods: The data presented represent the average change ± standard deviation for the 17 recordings. The value represents the difference between the first period and the second period in the title. For instance, in MTI-PRE, a positive value indicates an increase in the variable during MTI compared to PRE. Values are presented; p-values were calculated by permutation test and then corrected via false rate discovery (FDR). p-values < 0.05 indicated significant changes and were marked with an *.
